# C/EBP-β-regulated complement hyperactivation in spleen of SFTSV-infected mice: A clue to targeted complement therapy

**DOI:** 10.1371/journal.ppat.1014144

**Published:** 2026-04-15

**Authors:** Yan Liu, Meng Xu, Xihua Huang, Xiaohan Liu, Kai Fan, Yingying Zhang, Chen Xing, Hailin Tang, Jiayue Jiang, Kehan Xu, Maozhang He, Gang Xu, Cong Zhang

**Affiliations:** 1 State Key Laboratory for Safe Mining of Deep Coal Resources and Environment Protection, School of Public Health, Anhui University of Science and Technology, Huainan, China; 2 Department of Microbiology, School of Basic Medical Sciences, Anhui Medical University, Hefei, China; 3 Anhui Province Key Laboratory of Zoonoses, Anhui Medical University, Hefei, China; 4 Department of Traditional Chinese Medicine, The First Affiliated Hospital of Anhui Medical University, Hefei, China; 5 Department of Microbiology, Faculty of Naval Medicine, Navy Medical University, Shanghai, China; 6 The Second Clinical Medical School, Anhui Medical University, Hefei, China; 7 Department of Infectious Diseases, The First Affiliated Hospital of Anhui Medical University, Hefei, China; 8 Department of Infectious Diseases, The Second Affiliated Hospital of Anhui Medical University, Hefei, China; UTMB: The University of Texas Medical Branch at Galveston, UNITED STATES OF AMERICA

## Abstract

SFTSV, a tick-borne bunyavirus that causes severe fever with thrombocytopenia syndrome (SFTS), exhibits symptoms such as thrombocytopenia, leukocytopenia, hemorrhage, disseminated intravascular coagulation, and even multiple-organ failure. Nevertheless, the underlying pathogenic pathways of this disease remain poorly understood. In this study, by utilizing a lethal mouse model of *IFNAR*^*−/−*^ mice, we discovered that complement hyperactivation occurs in the spleen, and the spleen serves as a source of the central complement component C3 in SFTSV-infected mice. Infection of spleen stromal fibroblastic reticular cells with SFTSV significantly induces intracellular complement pathways through a type I IFN-independent manner and generates the active component C3a through the alternative pathway. The transcription of *C3* and complement factor B (*Cfb*) in SFTSV-infected fibroblastic reticular cells is regulated by C/EBP-β. Administration of a C3aR antagonist leads to a reduction in virus load and the inflammatory response, alleviates spleen injury, and prolongs the survival time in *IFNAR*^*-/-*^ mice. Collectively, our findings suggest that targeted complement therapy holds potential for the intervention of severe SFTS.

## Author summary

SFTS has high mortality and threatens public health significantly. Reportedly, compared to survivors, deceased SFTS patients show greater overactivation of the complement cascade. Nevertheless, how SFTSV induces complement activation and what role it plays in the pathogenesis of SFTS has not been clearly elucidated. In our study, we found that in SFTSV-infected mice, complement hyperactivation occurs in the spleen, which serves as a source of complement C3. In our in vitro experiments using SFTSV-targeted spleen fibroblastic reticular cells, we demonstrated that SFTSV infection activates intracellular complement pathways regulated by C/EBP-β, thereby generating activated C3a through the alternative pathway. When we administered a C3aR antagonist, we observed a reduction in virus load and the inflammatory response; which alleviated spleen injury and extended the survival time of *IFNAR*^*-/-*^ mice. Our findings suggest that targeted complement therapy may be a promising strategy for severe SFTS.

## Introduction

Severe fever with thrombocytopenia syndrome virus (SFTSV), also known as *Dabie bandavirus*, is a member of the *Phenuiviridae* family and can cause severe fever with thrombocytopenia syndrome (SFTS) [1]. Due to the lack of approved vaccines and specific therapeutics, the mortality rate of SFTS has remained relatively high, up to 30%. Patients with SFTS present clinical manifestations including high fever, thrombocytopenia, leukocytopenia, hemorrhage, disseminated intravascular coagulation (DIC), and even multiple-organ failure [2,3]. However, the pathogenic mechanism of the disease is poorly understood.

The complement system is one of the evolutionarily conserved components of innate immunity and has been shown to contribute to pathogen recognition and removal [4,5]. When the host is infected with pathogens, the complement system is activated via the classical, lectin, and alternative pathways. Complement component 3 (C3) can be activated through all three pathways into its active fragments C3a and C3b by C3 convertases. C3b generation is the amplification step that triggers downstream complement events, along with the generation of C5a and the membrane attack complex. C3a and C5a are anaphylatoxins. By signaling through their respective receptors, they can increase vascular permeability, induce the expression of proinflammatory cytokines, and are associated with DIC [6–9].

Abnormal complement activation has been reported to contribute to pathogenesis of severe illness induced by H5N1, SARS-CoV, and SARS-CoV-2 [10–13]. A recent study suggested it may also contribute to the pathogenesis of SFTS. Deceased SFTS patients were reported to have a higher level of complement cascade overactivation compared to survivors [14]. However, the mechanisms of SFTSV-induced complement activation and its role in the pathogenesis of SFTS are currently unclear.

Here, we examined complement activation in *IFNAR*^*−/−*^ mice, a lethal mouse model of SFTS [15]. The complement system was one of the most highly induced intracellular pathways in response to SFTSV infection, and C3 was processed into active products in spleen stromal fibroblastic reticular cells (FRCs). Intracellular complement C3 activation was regulated by C/EBP-β, which is closely related to SFTSV replication and the regulation of innate and adaptive immunity. Administration of a C3a receptor antagonist decreased virus load and the inflammatory response, and attenuated spleen injury in *IFNAR*^*−/−*^ mice.

## Result

### Complement activation occurs in the spleen of SFTSV-infected *IFNAR*^*−/−*^ mice

**Fig 1:**
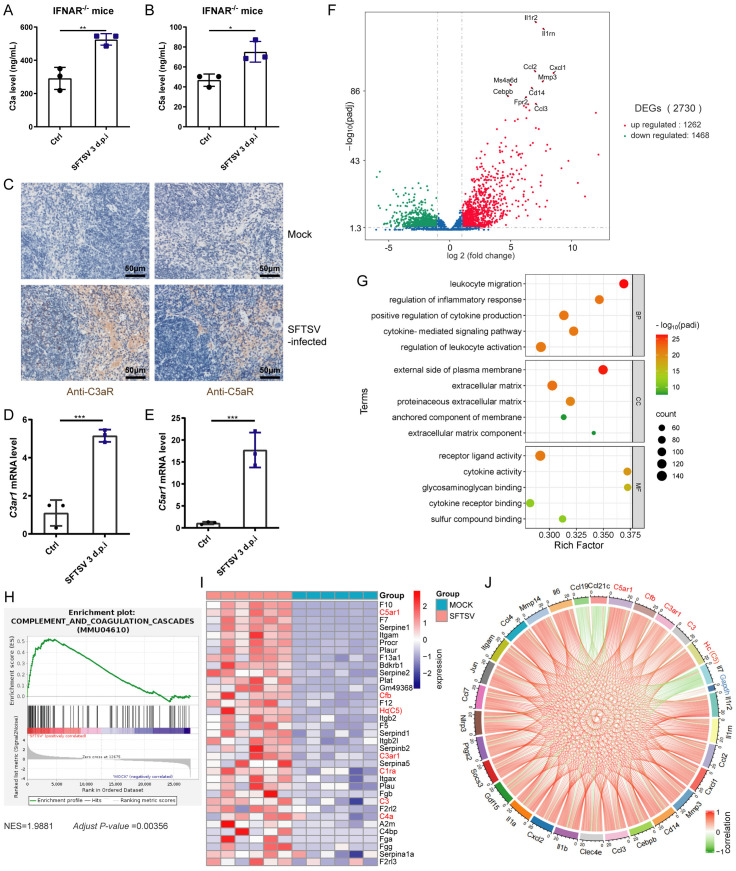
SFTSV infection induces complement activation in the spleen of IFNAR^−/−^ mice. **(A and B)** Serum C3a (A) and C5a (B) levels of *IFNAR*^*^−/−^*^ mice infected with SFTSV for 3 days or mock-infected. Data are presented as the mean ± SD. **(C)** Immunohistochemical staining for C3aR and C5aR in the spleen. The deposition of C3aR and C5aR significantly increased 3 days after SFTSV infection. Scale bars are indicated in the images. **(D and E)** *C3ar1* (D) and *C5ar1* (E) mRNA expression levels in the spleen 3 days post-infection. Data are presented as the mean ± SD. **(F)** Volcano plot indicating DEGs in mock-infected and SFTSV-infected spleens. Upregulated and downregulated genes with FDR < 0.05 and fold change > 2 are colored red and green, respectively. **(G)** Gene ontology (GO) analysis based on DEGs in SFTSV-infected spleens (FDR < 0.05 and fold change > 2). **(H)** Gene set enrichment analysis (GSEA) plot for the complement and coagulation cascades pathway. **(I)** Heatmap showing the expression profiles of genes involved in the complement and coagulation cascades pathway, with complement genes highlighted in red. **(J)** Circos graph displaying the correlation network between complement genes and the top 25 DEGs associated with the most enriched biological processes in **(G)**; the *Gapdh* gene was added as a reference. Each sector of the circle represents one gene, and the color of each link represents the Spearman correlation coefficient between the expressions of genes. Two-sided *p*-values, examined by Student’s t-test (A, B, D and **E)**, are shown. *p < 0.05, **p < 0.01, ***p < 0.001.

To determine whether complement activation is involved in the pathogenesis of SFTSV, we detected anaphylatoxins C3a and C5a and their respective receptors in the blood and multiple tissues of *IFNAR*^*−/−*^ mice. Serum C3a and C5a levels were significantly increased in SFTSV-infected mice (Fig 1A and 1B). Deposition of C3a receptor (C3aR) and C5a receptor (C5aR) was mainly observed in the red pulp of the spleen in SFTSV-infected mice (Fig 1C). To confirm the immunohistochemical observations, we analyzed the gene expression of C3aR and C5aR in spleen tissues using quantitative real-time PCR. The mRNA expression levels of complement component 3a receptor 1 (*C3ar1)* and complement component 5a receptor 1 (*C5ar1)* were significantly increased in the spleens of SFTSV-infected mice (Fig 1D and 1E). The deposition of C3aR and C5aR was barely detectable in the heart, lung, liver, and kidney tissues (S1A and S1B Fig). These data indicate that complement is activated in SFTSV-infected mice, particularly in the spleen.

The spleen is a major secondary immune organ, a filter for blood in the immune system, and also a primary replication site of SFTSV [15,16]. To obtain a comprehensive understanding of the pathogenic mechanisms of SFTSV infection, we conducted transcriptomic analysis of splenic tissues. Principal component analysis (PCA) revealed that the targeted transcriptomic profiles clearly distinguished the spleens of SFTSV-infected mice from those of mock-infected mice (S1C Fig). In total, 2730 genes were identified as having significantly different expression levels between the two groups (fold change > 2 and false discovery rate (FDR) < 0.05), with 1468 genes downregulated and 1262 genes upregulated in the SFTSV-infected group (Fig 1F). Gene ontology (GO) analysis indicated that the most strongly regulated biological processes were related to “leukocyte migration,” “regulation of inflammatory response,” and “positive regulation of cytokine production” (Fig 1G). Notably, through gene set enrichment analysis (GSEA) for pathway enrichment, the KEGG “complement and coagulation cascades” pathway was also significantly upregulated after SFTSV infection (Fig 1H). The expression levels of *C3ar1*, *C5ar1*, *C3*, complement component 5 (*C5*), and complement factor B (*Cfb*) were significantly induced by SFTSV infection (Fig 1I). Correlation analysis revealed a significant correlation between the expression of these complement genes and the differentially expressed genes (DEGs) associated with the most enriched biological processes (Fig 1J). These results collectively indicate that complement activation is potentially involved in the pathogenesis of SFTSV infection in the spleen.

### SFTSV infection induces complement C3 production in the spleen

**Fig 2:**
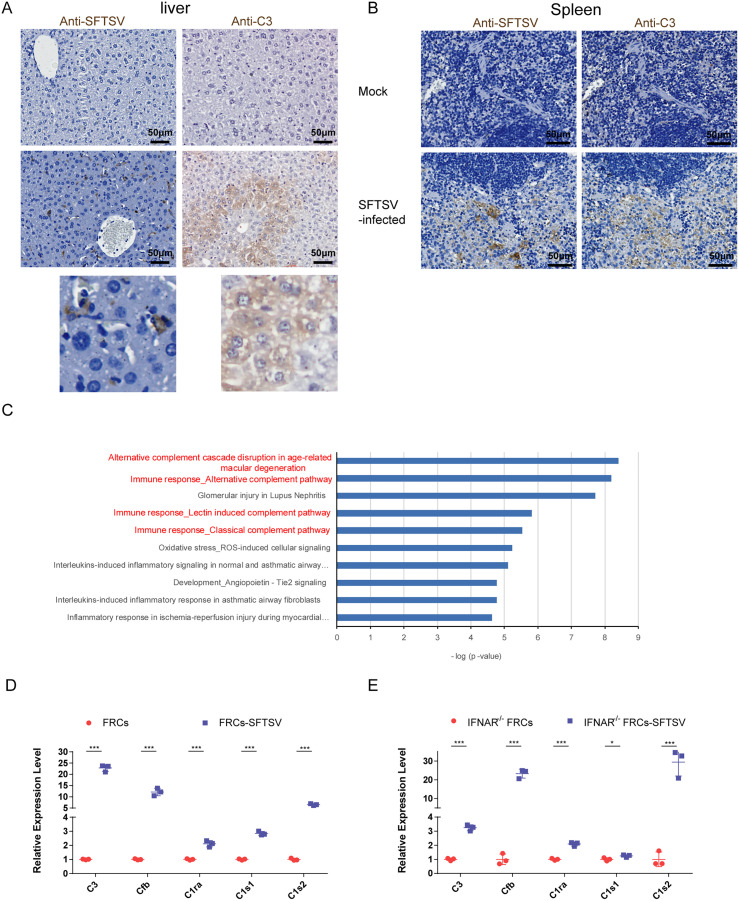
SFTSV infection induces complement C3 production in the spleen of *IFNAR*^*−*^^*/*^^*−*^ mice. **(A)** Mice were sacrificed, and tissues were collected 3 days after SFTSV infection. Immunohistochemical staining for C3 and SFTSV was performed on the liver tissues. Local areas of the images, which showed SFTSV-positive and C3-positive cells, was magnified to provide a clearer view. **(B)** Serial sections were prepared for immunohistochemical staining to detect SFTSV and C3 in the spleen. Representative images of immunohistochemical staining from ≥ 3 independent experiments are shown. Scale bars are indicated in the images. **(C)** The top 10 significantly enriched pathways analyzed by Metacore using DEGs between SFTSV-infected and mock-infected FRCs. **(D and E)** The mRNA expression levels of complement-related genes, including *C3, C1ra, C1s1, C1s2,* and *Cfb* in WT FRCs (D) or *IFNAR*^*−/−*^FRCs (E) infected with SFTSV. Data are presented as the mean ± SD. Two-sided *p*-values, examined by Student’s t-test, are shown. *p < 0.05, **p < 0.01, ***p < 0.001.

As the traditional doctrine, complement is viewed as a hepatocyte-derived, systemic serum effector system for pathogen recognition and removal [17,18]. C3 is a central component of the complement system during its activation [19]. In order to examine the sources of C3 following SFTSV infection, we performed immunohistochemical staining of C3 in the spleen, heart, lung, liver, and kidney tissues. High levels of C3 were mainly observed in the spleen and liver tissues of SFTSV-infected mice, while it was not detected in the heart, lung, and kidney tissues (Figs 2A, 2B and S2A). In the liver tissues of infected mice, C3 deposition was located in hepatocytes and SFTSV antigen was located in non-hepatocytes (Fig 2A). In the spleen tissues, the deposition of C3 significantly increased in the location of viral antigen-positive cells (Fig 2B), suggesting that elevated C3 might be derived from SFTSV-target cells in the spleen, in addition to hepatocytes.

Fibroblastic reticular cells (FRCs) are the main stromal cells of secondary lymphoid organs, generate the physical scaffold, and produce chemokines, cytokines, and growth and survival factors to regulate innate and adaptive immunity [20]. Previous studies reported that FRCs appear to be one of the major targets of SFTSV in the spleen tissues [15,21]. We isolated primary FRCs from the spleen tissues of wild-type and *IFNAR*^*−/−*^ mice and established immortalized reticular cell lines as previously described [22]. We then performed high-throughput RNA sequencing on FRCs infected with SFTSV. Using DEGs after SFTSV infection and the Metacore database for GO analysis, we found that the most significant biological process terms corresponded to “immune responses” and “vascular endothelial cell damage” (S2B Fig). Complement pathways were among the most enriched pathways ranked by significance, four of which were among the top 5 most significantly induced pathways in FRCs (Fig 2C). Gaining insight into the DEGs in the complement pathways, we noted that transcription of genes in both the classical complement pathway and the alternative complement pathway was highly activated, including components of the C1 proteases (*C1ra*, *C1s1* and *C1s2*), *Cfb*, and complement *C3* (Fig 2D). Previous studies have shown a potential direct crosstalk between interferon (IFN) and complement activation [14,23,24]. Several complement cascade genes, including *C3*, *Cfb*, complement component 2 (*C2*) and complement C4B (*C4b*), were identified as IFN-stimulated genes (ISGs) [23]. However, it seems complement activation in SFTSV**-**infected FRCs is independent of type I IFN responses, because *IFNAR*^*−/−*^ mice have high levels of complement activation. We also performed RNA sequencing on *IFNAR*^*−/−*^ FRCs. Complement pathways were again highly enriched after SFTSV infection, and the transcription of complement cascade genes, such as *C3*, *C1ra*, *C1s1*, *C1s2,* and *Cfb*, was also significantly increased (Fig 2E). Collectively, these data suggest that the complement system is one of the remarkably activated processes within SFTSV-infected FRCs, and this activation occurs through a type I IFN-independent pathway.

### C3 is processed into active product C3a in SFTSV-infected FRCs

**Fig 3:**
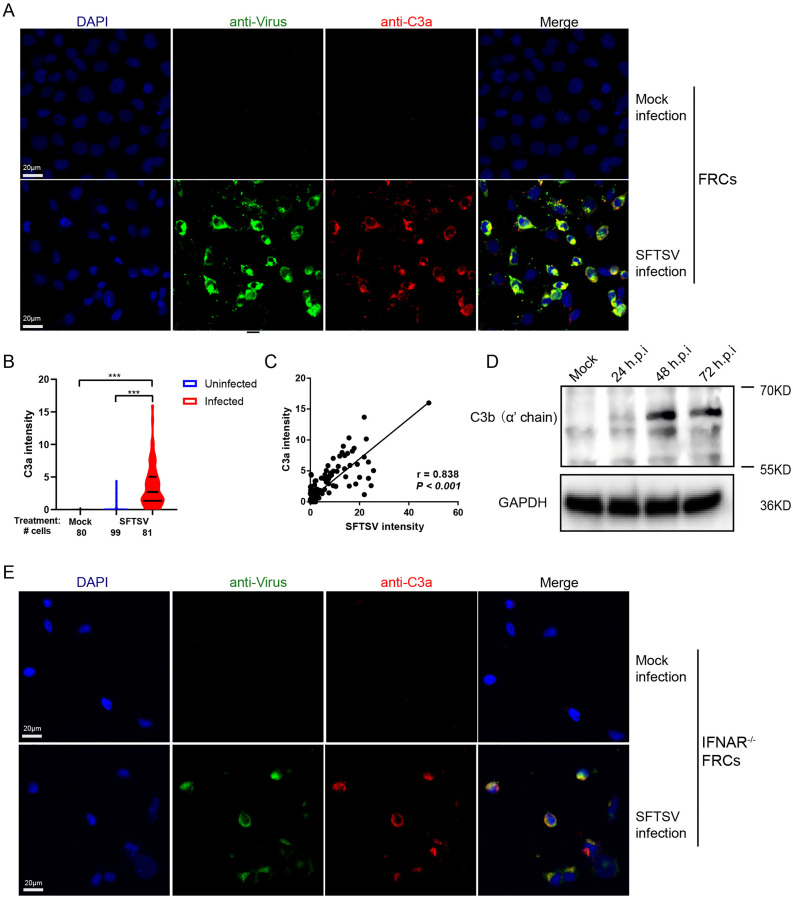
SFTSV infection induces C3 cleavage in FRCs. Representative confocal images from 3 independent experiments showing the expression of C3a and SFTSV antigen in SFTSV-infected or mock-infected FRCs. **(B)** Quantification of the C3a fluorescence signal in SFTSV-infected or mock-infected FRCs. The number of cells is indicated below each violin. *p < 0.05, **p < 0.01, ***p < 0.001, by Dunnett’s multiple comparisons test after ordinary one-way ANOVA. **(C)** Correlation between the C3a fluorescence signal and the SFTSV antigen fluorescence signal on a per-cell basis in FRCs. Indicated are the Pearson correlation coefficients and the associated *p*-value. **(D)** The expression of C3b in FRCs at the indicated times after SFTSV infection. **(E)** Representative confocal images from 3 independent experiments showing the expression of C3a and SFTSV antigen in SFTSV-infected or mock-infected *IFNAR*^*−/−*^ FRCs. Scale bars are indicated in the images.

C3 cleavage by C3 convertase generates active fragments C3a and C3b. Elevated expression of *Cfb*, and *C3* in SFTSV-infected FRCs suggest the presence of the intracellular C3 convertase, which could cleave C3 into its active fragments (Fig 2D and 2E). To determine whether C3 was processed into the active C3a fragment in these cells, we infected FRCs with SFTSV and used confocal imaging to detect C3a expression. There was minimal C3a in mock-infected cells, and the intracellular C3a fluorescence signal was significantly higher in SFTSV-infected cells than in both mock-infected and uninfected cells (Fig 3A-3C). We also observed a direct linear correlation between C3a levels and the SFTSV antigen signal in FRCs (Fig 3C), suggesting a relationship between C3 activation in SFTSV-infected FRCs and viral load. C3 cleavage was additionally detected via western blotting, and the C3 cleavage fragment C3b was significantly upregulated following SFTSV infection (Fig 3D). Furthermore, both C5 and its cleavage fragment C5b were found to be upregulated in SFTSV-infected FRCs (S3A Fig). Notably, intracellular C3 and C5 cleavage was also validated in mouse embryonic fibroblasts (MEFs), an SFTSV-susceptible cell line (S3B and S3C Fig). We further performed confocal imaging to assess C3a in *IFNAR*^*−/−*^ FRCs; intracellular C3a fluorescence signals were also increased in SFTSV-infected cells and colocalized with viral antigen signals (Fig 3E), demonstrating that the C3 cleavage process is independent of type I IFN regulation. Collectively, these data demonstrate that FRCs are not only a source of complement C3 but also of its active fragment C3a, which may partly explain the more significantly C3aR signaling activation in the mouse spleen than in other tissues (Fig 1C).

### SFTSV infection induces intracellular C3 activation via the alternative complement pathway

**Fig 4:**
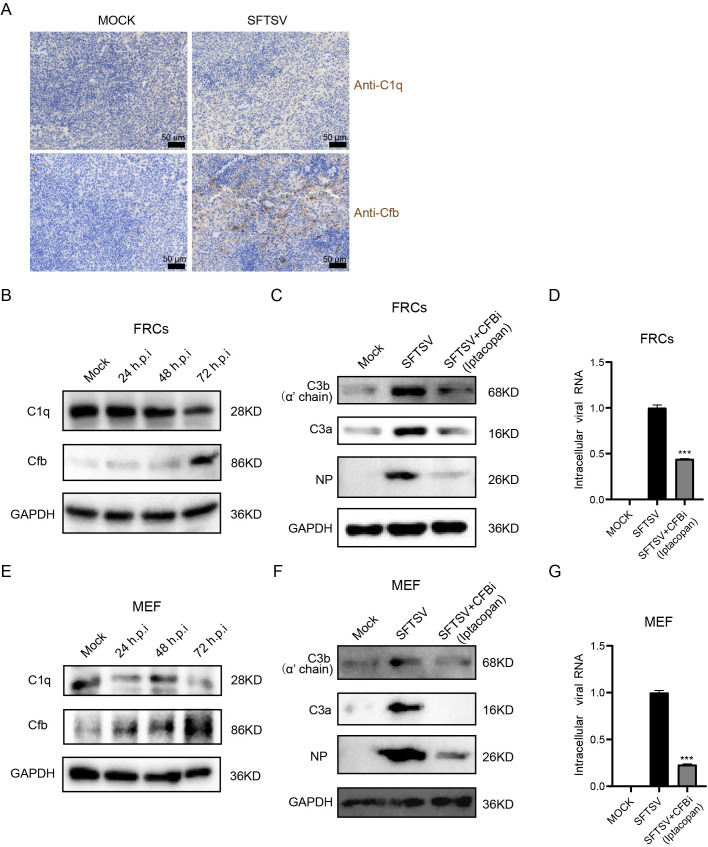
SFTSV induces intracellular C3 cleavage via the alternative complement pathway. Immunohistochemical staining for C1q and Cfb in the spleens. Mice were sacrificed and tissues were collected 3 days after SFTSV infection. **(B)** The expression levels of C1q and Cfb in FRCs at the indicated times after SFTSV infection (1.0 MOI) were detected by Western blotting. **(C)** FRCs were mock-infected or infected with SFTSV (MOI of 1, 72 hpi), with or without treatment of the CFB inhibitor Iptacopan (100 µM). Intracellular C3a, C3b, and viral NP protein levels were analyzed by Western blotting. **(D)** Intracellular viral RNA levels were quantified by RT‒qPCR. **(E)** The expression levels of C1q and Cfb in MEFs at the indicated times after SFTSV infection (1.0 MOI) were detected by Western blotting. **(F)** MEFs were mock-infected or infected with SFTSV (MOI of 1, 48 hpi), with or without Iptacopan treatment (100 µM). Intracellular C3a, C3b, and viral NP protein levels were analyzed by Western blotting. **(G)** Intracellular viral RNA levels were quantified by RT‒qPCR. Data are presented as mean ± SD. Two-sided *p*-values, examined by Student’s t-test, are shown. ***p < 0.001.

Complement C1, the initiator of the classical complement pathway, forms a macromolecular complex comprising C1q, C1r, and C1s. The classical pathway is activated upon binding of C1q to immune complexes, and then the activated C1 complex cleaves C2 and C4 to form the classical C3 convertase C4bC2b. Cfb assembles with C3b to form the alternative pathway C3 convertase C3bBb [25]. Transcriptomic sequencing of FRCs revealed elevated expression of the components of C1 proteases (C1r and C1s), as well as Cfb, following SFTSV infection, implying that SFTSV may trigger the complement activation via either the classical or alternative pathway. To determine which complement pathway is critical for SFTSV infection, we first examined the protein levels and localization of C1q (indispensable for classical pathway initiation) [25] and Cfb in the spleens of SFTSV-infected mice. Our results showed that Cfb, but not C1q, was predominantly deposited in the red pulp of the spleen in SFTSV-infected mice (Fig 4A). We further evaluated intracellular C1q and Cfb in SFTSV-infected FRCs and MEFs by western blotting; Cfb expression was significantly upregulated in a time-dependent manner following infection, whereas C1q protein levels were not increased and even slightly decreased (Fig 4B and 4E). These results indicate that the alternative pathway, rather than the classical pathway, contributes mainly to SFTSV infection-induced C3 activation. The upregulation of C1r and C1s is therefore likely to mediate functions independent of classical pathway activation. To further verify the key role of the alternative complement pathway, we used the selective CFB inhibitor Iptacopan to block alternative pathway activation in SFTSV-infected FRCs and MEFs, and subsequently assessed C3 cleavage and viral replication. Treatment of FRCs and MEFs with Iptacopan reduced the production of C3a and C3b after SFTSV infection to levels close to those in the mock-infected group (Fig 4C and 4F). Furthermore, western blot analysis of viral nucleoprotein (NP) and quantitative real-time PCR (qRT-PCR) of viral RNA both demonstrated that Iptacopan significantly inhibited SFTSV replication (Fig 4D and 4G). Collectively, these findings confirm that the alternative complement pathway is essential for SFTSV infection and plays a pivotal role in C3 cleavage and viral replication.

### *C3* and *Cfb* transcription in SFTSV-infected FRCs is regulated by C/EBP-β

**Fig 5:**
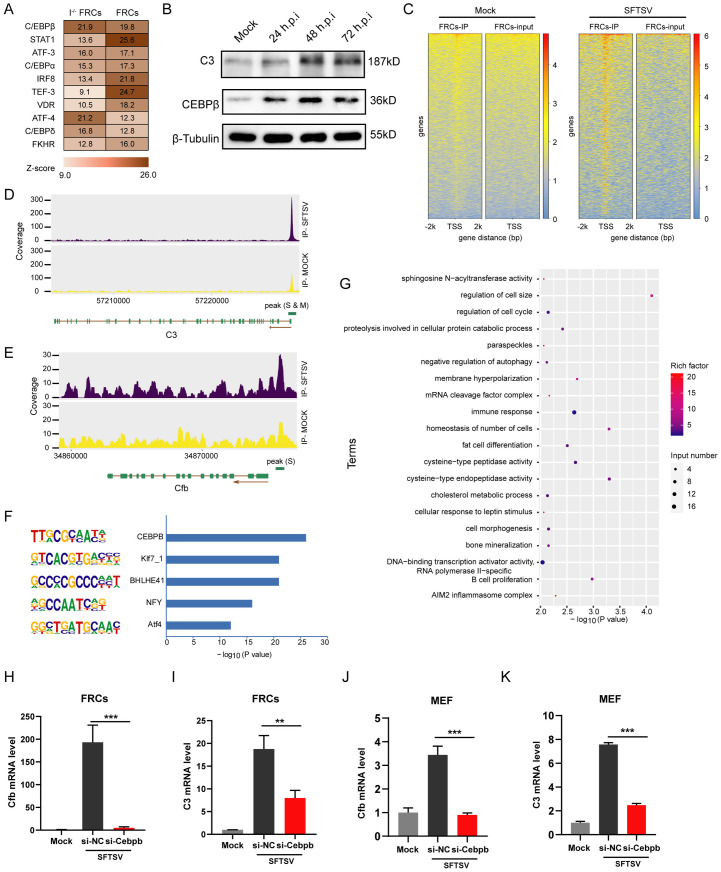
C/EBP-β transcriptionally regulates complement *C3* and *Cfb* expression induced by SFTSV infection. The top 10 TFs that were predicted by Metacore regulating the SFTSV-driven transcriptional response in WT and *IFNAR*^*−/−*^ FRCs. **(B)** The expression levels of C3 and C/EBP-β in FRCs at the indicated times after SFTSV infection. **(C)** FRCs were mock-infected or infected with SFTSV (MOI of 1, 72 hpi) for C/EBP-β ChIP-seq analysis. Distribution of normalized read counts in a 4,000 bp window around C/EBP-β-specific peaks in SFTSV-infected or mock-infected FRCs. **(D and E)** ChIP-seq tracks displaying the C3 (D) and Cfb (E) gene loci in SFTSV-infected versus mock-infected FRCs. A prominent peak is evident at the C3 promoter region in both groups, whereas a distinct peak is observed at the Cfb promoter only in the SFTSV-infected group. **(F and G)** The top 5 significant HOMER *de novo* motifs (F) and the top 20 significantly enriched GO terms (G) of different peaks in FRCs between SFTSV-infected and mock-infected samples. (H and **I)** The mRNA expression levels of *Cfb* (H) and *C3* (I) in FRCs with siRNA transfection. **(J and K)** The mRNA expression levels of *Cfb* (J) and *C3* (K) in MEFs with siRNA transfection. Two-sided *p*-values, examined by Student’s t-test, are shown. *p < 0.01, ***p < 0.001.

To gain insights into the transcriptional regulation of the activated complement system, we assessed the genes differentially regulated by SFTSV in FRCs and the *IFNAR*^*−/−*^ FRCs. We used Metacore to predict the transcriptional regulators with the up-regulated genes. Of the top 10 common transcription factors predicted in both cell lines, three belong to the CCAAT/enhancer binding protein (C/EBP) family, and the most enriched transcription factor was C/EBP-β (Fig 5A), which had been reported to be one of the transcription factors regulating the expression of *C3* gene [26]. Chromatin immunoprecipitation sequencing (ChIP-seq) datasets of C/EBP-β and H3K27Ac (a marker of active promoter and enhancer regions) sourced from the ENCODE database also provided evidence that C/EBP-β can bind to the promoter regions of complement-related genes including *C3*, *C1S,* and *CF*B (S4A Fig). We then analyzed the change in C/EBP-β expression after SFTSV infection in FRCs. The western blot assay showed that C/EBP-β expression was significantly upregulated from 24 hours post infection (hpi) and increased continuously at 48 hpi and 72 hpi (Fig 5B). C3 expression showed the same kinetics (Fig 5B). We also obtained RNA sequencing data of whole blood cells from SFTS patients (GSE144358). Transcriptome analysis showed that the expression levels of complement pathway-related genes, including *C1QA*, *C1QB*, *C1QC*, *CFB*, *C3*, and *C5*, and the transcription factor C/EBP-β, were remarkably upregulated and related to disease severity and prognosis (S4B and S4C Fig). Pearson correlation analysis showed that C/EBP-β expression levels correlated linearly with *C3* and *CFB* mRNA expression levels in patients’ peripheral blood (S4D Fig).

To further investigate the transcriptional regulation of C/EBP-β after SFTSV infection, we performed chromatin immunoprecipitation sequencing (ChIP-seq) of C/EBP-β in FRCs with SFTSV infection or mock infection. C/EBP-β peaks showed increased signals in FRCs infected with SFTSV relative to mock-infected FRCs (Fig 5C). In SFTSV-infected FRCs, C/EBP-β binding peaks were present at the promoter regions of both *C3* and *Cfb*, and the read coverage of these binding peaks is higher than that in mock-infected FRCs (Fig 5D and 5E). *De novo* motif analysis of the different peaks identified the binding motif of C/EBP-β as the most highly enriched (Fig 5F). The genes close to these peaks were related to GO terms for “immune response”, “B cell proliferation”, and “AIM2 inflammasome complex”, suggesting that C/EBP-β regulates the immune response in FRCs after SFTSV infection (Fig 5G). To validate whether C/EBP-β directly regulated complement genes in FRCs after infection, we knocked down C/EBP-β expression with small interfering RNAs (siRNA) and detected *C3* and *Cfb* mRNA expression. Indeed, the high expression levels of *C3* and *Cfb* induced by SFTSV infection were significantly decreased in FRCs transfected with C/EBP-β siRNA compared to those transfected with negative control (NC) siRNA (Fig 5H and 5I). We further validated this regulatory effect of C/EBP-β in MEFs, where SFTSV-induced *C3* and *Cfb* expression was also significantly reduced in C/EBP-β siRNA-transfected cells relative to NC siRNA-transfected controls (Fig 5J and 5K). Together, these data strongly suggest that the activation of complement C3 and Cfb in SFTSV-infected FRCs is transcriptionally regulated by C/EBP-β.

### C3a/C3aR signaling regulates SFTSV replication, vascular endothelial permeability, and inflammatory response in FRCs

**Fig 6:**
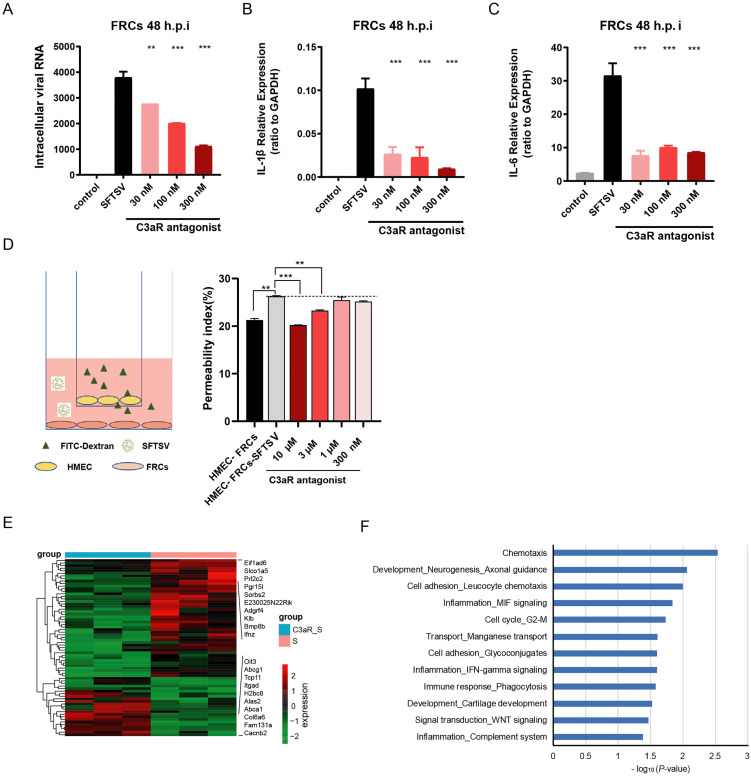
The cellular C3a/C3aR signaling pathway regulates SFTSV replication, inflammation in FRCs, and vascular endothelial permeability. **C)** Intracellular viral RNA (A) and the mRNA expression levels of cytokines IL-1β (B) and IL-6 (C) in SFTSV-infected FRCs treated with or without the C3aR antagonist (SB290157). **(D)** Schematic diagram of 40-kDa FITC-dextran extravasation analysis using a co-culture of FRCs and the endothelial cell line HMEC-1 (left) and the calculated permeability index in co-cultures treated with SFTSV or SFTSV plus the C3aR antagonist for 24 hpi. Data are presented as the mean ± SD. Two-sided *p*-values, examined Dunnett’s multiple comparisons test after one-way ANOVA, are shown. **p < 0.01, ***p < 0.001. **(E)** A heatmap showing the DEGs of SFTSV-infected FRCs in the presence of the C3aR antagonist or the vehicle. The top 10 upregulated and downregulated genes are highlighted. **(F)** Significantly enriched pathways analyzed by Metacore using the DEGs of SFTSV-infected FRCs in the presence of the C3aR antagonist or the vehicle.

SFTSV infection altered the immune response as well as vascular endothelial cell damage in FRCs, accompanied by abundant C3a production (Figs 3A and S2B). To explore the role of intracellular C3 activation in these processes, we assessed SFTSV replication, vascular endothelial cell permeability, and inflammatory response in SFTSV-infected FRCs with C3a/C3aR signaling inhibition. Q-PCR results of the viral RNA and *IL-1β* and *IL-6* mRNA showed that C3a/C3aR signaling inhibition via C3aR antagonist (C3aRA) induced a dose-dependent decrease in SFTSV replication and a comparable decrease in the expression of *IL-1β* and *IL-6* (Fig 6A-6C). An identical trend was observed in MEFs (S5A-S5C Fig). We used the FITC-dextran extravasation assay to assess the vascular endothelial cell integrity in an FRCs and HMEC-1 coculture model. SFTSV infection significantly increased the permeability of HMEC-1, and C3aRA decreased the permeability of HMEC-1 in a dose-dependent manner (Fig 6D). We also performed transcriptome sequencing of FRCs treated with C3aRA or the vehicle after SFTSV infection to obtain DEGs influenced by C3a/C3aR signaling (Fig 6E). Pathway analysis using these DEGs suggested that C3a/C3aR signaling impacted many important pathways, including “chemotaxis”, “cell adhesion-leucocyte chemotaxis”, “inflammation-MIF signaling”, and “immune response-phagocytosis”, which contribute to FRCs providing a microenvironment for the optimal immune cell activation and anti-viral response (Fig 6F). Taken together, our results indicated that intracellular C3 activation in FRCs was closely related to SFTSV replication, vascular endothelial permeability, and its function of regulating innate and adaptive immunity.

### C3aR antagonist decreased virus load, attenuated spleen injury, and reduced the inflammatory response in *IFNAR*^*-/-*^ mice after SFTSV challenge

**Fig 7:**
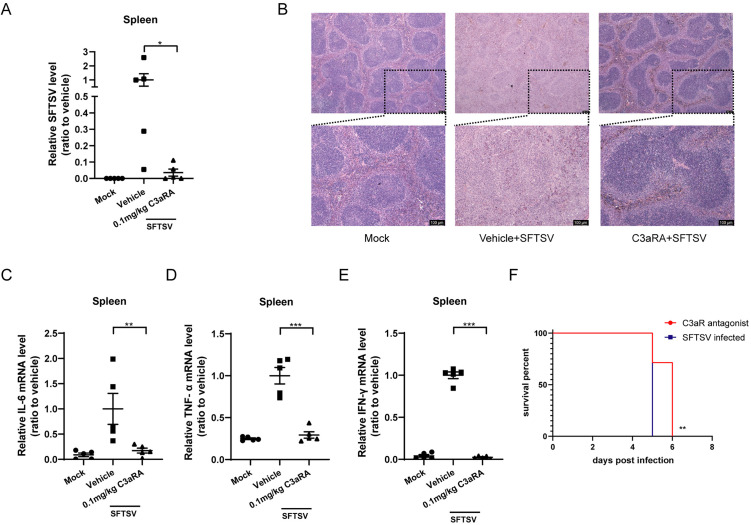
Complement inhibition using a C3aR antagonist is effective against SFTSV infection in *IFNAR*^*−*^^*/*^^*−*^ mice. *IFNAR*^*−/−*^mice were challenged with SFTSV and then administered the C3aR antagonist (C3aRA) or the vehicle control. Viral load in the spleen 3 days after challenge in the SFTSV + vehicle group (n = 5) and the SFTSV + C3aR antagonist group (n = 5). **(B)** Representative images of the histological analysis of the spleen tissues from mock-infected or SFTSV-infected mice administered the C3aRA or the vehicle 3 days post-infection. Scale bars are indicated in the images. **(C-E)** The mRNA expression levels of the cytokines IL-6, TNF-α, and IFN-γ in the spleen tissues 3 days post-infection (n = 5 per group). Data are presented as the mean ± SD. Two-sided p-values, examined by Student’s t-test, are shown. *p < 0.05, **p < 0.01, ***p < 0.001. **(F)** Kaplan-Meier survival curves of SFTSV-infected *IFNAR*^*−/−*^ mice treated with the C3aR antagonist or the vehicle (n = 6 per group). Representative data are shown from two independent experiments. **p < 0.01 (log-rank test).

We further blocked complement activation using C3aRA in *IFNAR*^*−/−*^ mice. Three days after infection, SFTSV replication was quantified by RT-PCR targeting the viral S segment. Compared with the SFTSV + vehicle group, virus titers were significantly lower in the SFTSV + C3aRA group in the spleen (Fig 7A). Gross damage and pathological changes in the spleen tissues were assessed by H&E staining. After SFTSV infection, severe spleen damage was observed, with evident loss of white pulp in the spleens of infected mice. Notably, in the SFTSV + C3aRA group, the destruction of white pulp was markedly attenuated (Fig 7B). We also measured the relative mRNA expression of IL-6, TNF-α, and IFN-γ to investigate whether complement activation is associated with increased inflammation. Decreased IL-6, TNF-α, and IFN-γ mRNA expression in the spleen (Fig 7C-7E), and other tissues including the liver, lung, kidney, and heart of C3aRA-treated mice indicated that C3aRA treatment attenuated systemic inflammation after SFTSV infection (S6 Fig). Additionally, we monitored the survival time of SFTSV-infected mice and found that C3aRA treatment significantly prolonged their survival (Fig 7F). Taken together, these results indicate that the inhibition of complement activation with C3aRA may be a novel remedy against SFTSV infection.

## Discussion

In this study, we showed that the induction of complement activation in the spleen is an SFTSV-driven event. C3 and Cfb were regulated by C/EBP-β in response to SFTSV in infected FRCs, and then C3 was processed into biologically active C3a via alternative pathway convertase. Inhibition of C3a/C3aR signaling could inhibit SFTSV replication, attenuate the SFTSV-induced inflammatory response *in vitro* and *in vivo*, and prolong the survival time of SFTSV-infected mice.

Although the soluble complement produced by the liver is a critical part of the protection against pathogens, the complement proteins and receptors are also expressed in immune cells, including monocytes, macrophages, and T-lymphocytes, as well as in non-immune cells, including epithelial cells, fibroblasts, and astrocytes [27]. While serum-circulating complement protects against invading pathogens, intracellularly active complement has recently been discovered and functions as a vital regulator of cell-metabolic events in immune cells and non-immune cells. Liszewski *et al.* found that complement activation occurred intracellularly in T cells and functionally regulated T cell homeostasis and effector differentiation [28]. Niyonzima *et al.* reported that monocytes and macrophages constitutively express C5 and generate active, autocrine C5a, which, through C5aR1 signaling on mitochondrial membranes, regulates IL-1β production underlying sterile inflammation in macrophages [29]. Yan *et al*. demonstrated that SARS-CoV-2 infection induced activated C3a production via an inducible alternative pathway convertase in respiratory epithelial cells, which acted on their cognate receptors to trigger local hyperinflammation in the lungs of COVID-19 patients [10]. Our results of IHC staining of C3 in multiple tissues of SFTSV-infected mice showed that, except for abundant deposition in liver tissues, the C3 level also significantly increased in antigen-positive cells in the mouse spleen. The complement system was one of the most highly induced intracellular pathways after SFTSV infection in spleen stromal cells and was transcriptionally regulated by C/EBP-β. Infection of FRCs with SFTSV generated activated complement component C3a. Considering that our results of the inhibition of C3a/C3aR signaling via a C3aR antagonist decreased SFTSV viral load and proinflammatory cytokines, intracellular complement activation might play an important role in local complement hyperactivation and in regulating viral replication and the immune response of the spleen.

Complement activation has been implicated in the pathogenesis of multiple viral infections and is related to critical illness. Previous studies have reported that the inhibition of complement activation alleviates H5N1-induced acute lung injury [12]. Relative to C57BL/6J control mice, SARS-CoV-infected C3^-/-^ mice exhibited significantly reduced lung pathology and lower cytokine and chemokine levels [13]. Inhibition of the lectin pathway of complement activation reduces the severity of acute respiratory distress syndrome in a mouse model of SARS-CoV-2 infection [11]. As for SFTS, a previous study measured 12 complement components in an SFTS case cohort and reported that the secretions of C3b/iC3b and complement factor D was remarkably induced in the deceased SFTS patients [14]. Additionally, a specific interest in SFTS stems from features of endothelial injury, disseminated intravascular coagulation, and cytokine storms, which are related to complement activation, suggesting that complement inhibition might be an alternative treatment for severe SFTS [6,8,10]. In this study, our results indicated that the inhibition of complement C3a/C3aR signaling attenuated SFTSV replication, reduced vascular endothelial permeability, and alleviated the inflammatory response. Although in the interferon receptor knockout mouse model, due to the intense response following SFTSV infection and the difficult-to-reverse fatal outcome, the C3aR antagonist failed to increase the survival rate of the mice, it extended their survival time. Targeted complement therapy still holds potential worthy of in-depth exploration for intervening in severe SFTS.

Despite these findings, this study has several limitations. First, the liver constitutes another major source of circulating complement proteins, and the relative contribution of splenic- versus hepatic-derived complement proteins to the elevated circulating complement protein levels remains undetermined. Although C3a levels are also increased in the circulation of SFTSV-infected mice and correlate with systemic inflammatory responses, the extent to which intracellular complement activation contributes to this elevation is unclear. Conditional deletion of *C3* in splenic or hepatic tissues may help further elucidate the tissue-specific roles of complement and intracellular complement activation in SFTSV infection. Furthermore, the specific SFTSV viral protein(s) responsible for complement hyperactivation, as well as the underlying mechanisms by which C3a/C3aR signaling activation promotes viral replication, requires further investigation.

## Materials and methods

### Ethics statement

The animal experiments in this work were approved by the Ethics Committee of Anhui Medical University, and they adhered to the Chinese National Guidelines for the Care of Laboratory Animals and the institutional animal care protocols.

### Cells and virus

The cell line of FRCs established in our laboratory was used in these experiments. Briefly, FRCs was isolated from the mouse spleen, purified by the fluorescence-activated cell sorting method, and immortalized with simian virus 40 [22]. FRCs and MEFs were cultured in DMEM medium (Gibco, USA) supplemented with 10% heat-inactivated fetal bovine serum (FBS) (ScienCell, USA), 100 U/mL penicillin, and 100 U/mL streptomycin (ScienCell, USA) at 37°C. HMEC-1 cells (Zqxzbio, China) were maintained in ECM medium (ScienCell, USA), supplemented with 10% FBS, 100 U/mL penicillin, and 100 U/mL streptomycin. SFTSV (strain AH001) was used in both *in vivo* and *in vitro* experiments. The virus was propagated in Vero cells, and the focus-forming assay was used to detect virus titers. All *in vitro* experiments involving live virus were performed in enhanced biosafety level 2 facilities, and all *in vivo* experiments were performed in animal biosafety level 3 facilities following governmental guidelines.

### Enzyme-linked immunosorbent assay (ELISA)

*IFNAR*^*−/−*^ mice were infected with 10^4^ PFU of SFTSV or mock-infected. Three days after infection, mouse serum was obtained. The levels of serum C3a and C5a was analyzed using the Mouse C3a ELISA kit (CUSABIO, China) and the Mouse C5a ELISA kit (CUSABIO, China), respectively, according to the manufacturer’s instructions.

### Immunohistochemistry

Spleen sections from *IFNAR*^*−/−*^ mice infected with 10^4^ PFU of SFTSV or mock-infected mice were used. At the indicated times in the figures, mice were sacrificed and spleen tissues were collected. Tissues were fixed in 10% formalin, and then embedded in paraffin. Five-micrometer serial sections were prepared for SFTSV and C3 deposition staining using a rabbit anti-SFTSV primary antibody and a rabbit anti-C3 primary antibody (Abcam, Cat. No. ab2000999, UK), respectively. Staining for C3aR, C5aR1, C1q, and Cfb was performed using a rabbit anti-C3aR (FineTest, Cat. No. 822006, China), a rabbit anti-C5aR1 (FineTest, Cat. No. FNab01118, China), a rabbit anti-C1q (Proteintech, Cat. No. 16889–1-AP, China), and a rabbit anti-Cfb (Proteintech, Cat. No. 84101–5-RR, China) primary antibody, respectively.

### Immunofluorescence

FRCs, *IFNAR*^*−/−*^ FRCs, and MEFs were infected with 1.0 multiplicity of infection (MOI) of SFTSV or mock-infected. At 72 hours post-infection, the cells were fixed, permeabilized, and blocked. For the co-staining of C3a and the virus, the C3a (Abcam, ab11873, UK) antibody and the HB29NP antibody were used as primary antibodies. The fluorescence of the cells was detected using a confocal microscope (LSM880, Zeiss, Germany) and analyzed using ImageJ v1.8.0.

### RNA sequencing and data analysis

FRCs and *IFNAR*^*−/−*^ FRCs were infected with SFTSV at an MOI of 1 or mock-infected. Total RNA was isolated using TRIzol reagent (Sigma-Aldrich) 72 hours post-infection. High-throughput RNA-seq was performed using the MGISEQ-2000 platform (with paired-end reads of 100 bp and a data amount of 10 GB for each sample). Bowtie2 (version 2.1.0) and Tophat2 (version 2.0.11) were used to map RNA-seq reads to the mouse genome (version mm10). DESeq2 software was used to assemble transcription units, calculate Fragments Per Kilobase of transcript per Million mapped fragments (FPKM) values, and identify differentially expressed genes (DEGs). For the transcriptomic analysis of whole blood cells from SFTS patients, we obtained data from GSE144358 and retrieved normalized gene expression levels (FPKM values). Metacore software (Clarivate Analytics, USA) was used to analyze functionally enriched pathways for DEGs. Upregulated DEGs in FRCs and IFNAR^−/−^ FRCs were used for transcription factor analysis using Metacore software.

### ChIP-seq data analysis

FRCs were infected with SFTSV (1 MOI) or mock-infected for 72 h. Then, the cells were fixed with 1% formaldehyde for 15 min and quenched with 0.125 M glycine. Genomic DNA regions of interest were isolated using an antibody against C/EBP-β (Abcam, ab32358, UK). ChIP assays were performed by SEQHEALTH Co., Ltd., Wuhan, China. DNA libraries were sequenced on the MGISEQ-T7 (paired-end, 300 bp) and the raw FASTQ files were aligned to the mouse reference genome (GRCm38/mm10). Macs2 was used to call C/EBP-β peaks and Csaw was used for differential peak analysis (SFTSV infection versus mock infection). HOMER was used to perform de novo motif analysis on differentially enriched peaks. H3K27Ac ChIP-seq (ENCFF706HLO) and CEBPB ChIP-seq (ENCFF000XBM) in HeLa cells were obtained from ENCODE, and the preprocessed and author-provided peak files (ENCFF144EOJ and ENCFF002CSA) were downloaded. ChIP-seq tracks were visualized using the IGV browser (Broad Institute).

### Quantitative real-time PCR

Total RNA from cells or mouse tissues was isolated using the TRIzol reagent (Sigma-Aldrich) following the manufacturer’s instructions. To analyze gene mRNA expression levels, cDNA was transcribed from 0.5 µg of total RNA using random primers and the PrimeScript RT Master Mix (RR036A, Takara, Japan). TB Green Premix Ex Taq II (Tli RNaseH Plus) (RR820A, Takara, Japan) was used for RT-qPCR on the LightCycler 480 PCR System (Roche, Switzerland). The relative gene expression levels were calculated using the ΔCt method and normalized to the mouse *Gapdh* gene. Detailed sequence information of the primers is shown in S1 Table.

### Western blotting analysis

FRCs and MEFs were collected at the indicated time points after SFTSV infection with RIPA lysis buffer. CFB inhibitor Iptacopan (TOPSCIENCE, China) was administered 2 h after SFTSV infection. The protein expression levels were detected by western blotting analysis following the protocol described previously [30]. The primary antibodies against C3 (Abcam, ab2000999, UK), C3/C3b/C3c (Proteintech, 21337–1-AP, China), C5/C5b (Affinit, DF7719, China), C1q (Proteintech, 16889–1-AP, China), Cfb (Proteintech, 84101–5-RR, China), CEBPB (Abcam, ab32358, UK) and β-tubulin antibodies (Proteintech, 10094–1-AP, China) and HRP-conjugated secondary antibodies (Apexbio) were used in this study. Protein expressions were detected using a Chemiluminescence imager (Gel Logic 2200PRO, Carestream, USA) and the Super ECL Plus kit (S6009M, US EVERBRIGHT, China).

### Small interfering RNA (siRNA)

siRNA targeting mouse *Cebpb* was purchased from GENERAL BIOLOGY (Shanghai, China), and the detailed sequence information of siRNAs was shown in S1 Table. FRCs and MEFs were plated in 12-well plates and transfected with 50 nM siRNA using Lipofectamine RNAiMAX reagent (Invitrogen, CA, USA). At 24 h after transfection, FRCs and MEFs were infected with SFTSV (1.0 MOI) or mock-infected. At 48 h after infection, cell samples were collected for further mRNA expression analysis or testing of gene knockdown efficiency.

### Animal experiments

*IFNAR*^*−/−*^ C57BL/6 mice (6–8 weeks old) were divided into three groups (n = 5 per group for tissue collection and n = 7 per group for survival rate): mock-infected, SFTSV + vehicle group, and SFTSV + C3aR antagonist (C3aRA, SB290157, TOPSCIENCE) group. For the SFTSV + vehicle and SFTSV + C3aR antagonist groups, mice were infected intraperitoneally with 10^4^ PFU of SFTSV; the mock-infected group received an equal volume of PBS via the same route. At 1 h, 24 h, and 48 h post-infection, mice in the SFTSV + C3aR antagonist group were administered intraperitoneally C3aRA at a dose of 0.1 mg/kg per mouse, while those in the SFTSV + vehicle and mock-infected groups were given an equal volume of vehicle via the same injection route. To measure the survival rate, the survival of the mice in each group was assessed daily for 10 days. The log-rank test was used to compare the Kaplan-Meier survival curves. To assess the virus load and the inflammatory response, mice were sacrificed 3 days after viral infection, and spleen tissues were collected. Half of the removed mouse spleen tissues were fixed in 4% paraformaldehyde, embedded in paraffin, and then hematoxylin and eosin (H&E) staining was performed for histological analysis

### Statistical analysis

Differences between groups were assessed for significance using the unpaired t-test, the Mann-Whitney U-test, or analysis of variance (ANOVA). Pearson correlation analysis was used for linear correlation fitting. Statistical analysis was performed using GraphPad Prism 8.0 version. The differences were considered statistically significant when the *p*-value < 0.05.

## Supporting information

S1 Fig Complement activation in tissues of IFNAR^−/−^ mice. (A and B) Immunohistochemical staining for C3aR (A) and C5aR (B) in the liver, kidney, heart, and lung tissues. Mice were sacrificed and tissues were collected 3 days after SFTSV infection. (C) PCA of transcriptomics data in mock-infected (n = 6) and SFTSV-infected (n = 6) spleens.
(TIF)

S2 Fig Complement C3 deposition in multiple tissues after SFTSV infection in IFNAR^−/−^ mice. Immunohistochemical staining for C3 in the kidney, heart, and lung tissues. Representative images of immunohistochemical staining from ≥3 independent experiments are shown. (B) Significantly enriched biological processes analyzed by Metacore using DEGs between SFTSV-infected and mock-infected FRCs. The Enrichment Map application in Cytoscape was used for visualization. Nodes represent biological process terms. The color and size of the nodes reflect the significance of the terms and the number of objects enriched in the terms, respectively. The connection between terms is based on shared objects.
(TIF)

S3 Fig SFTSV infection induces C3 cleavage in MEFs. Time-course expression of C5b in FRCs at the indicated time points following SFTSV infection. (B) Time-course expression of C3b and C5b in MEFs at the indicated time points following SFTSV infection. (C) Representative confocal microscopy images showing the expression of C3a and SFTSV antigen in SFTSV-infected or mock-infected MEFs. n = 3 independent experiments. Scale bars are indicated in the images.
(TIF)

S4 Fig Involvement of C/EBP-β in transcriptional regulation of complement pathways induced by SFTSV infection. C/EBP-β and H3K27Ac ChIP-seq tracks showing the C3, C1S, and CFB gene loci. ChIP-seq data were sourced from ENCODE (H3K27Ac and C/EBP-β). (B-D) Transcriptome data of peripheral blood mononuclear cells (PBMCs) from healthy controls and SFTS patients were sourced from GSE144358. SFTS patients were stratified into three groups according to their clinical status. “Recover”: SFTS patients in the convalescent phase, who had recovered from acute viral infection; “AcuteRecover”: SFTS patients in the acute phase of viral infection who ultimately achieved recovery; and “AcuteDeceased”: SFTS patients in the acute phase of viral infection who eventually succumbed to SFTSV infection. (B) The mRNA expression levels of complement-related genes, including *C1QA*, *C1QB*, *C1QC*, *C3*, *CFB*, and *C5*, in PBMCs from healthy controls and SFTS patients. (C) C/EBP-β mRNA expression in PBMCs from healthy controls and SFTS patients. Box-and-whiskers plot features: whiskers represent the lowest and greatest values; boxes represent the median with the 25th percentile and the 75th percentile. (D) Correlation between the mRNA expression of *C3* and *CFB* and the mRNA expression of C/EBP-β in PBMCs from healthy controls and SFTS patients. Indicated are the Pearson correlation coefficients and the associated p-values. Two-sided p-values, examined by Tukey’s multiple comparisons test after one-way ANOVA (B and C), are shown. *p < 0.05, ***p < 0.001.
(TIF)

S5 Fig The cellular C3a/C3aR signaling pathway regulates SFTSV replication and inflammatory responses in MEFs. (A-C) MEFs were mock-infected or SFTSV-infected (1.0 MOI, 48 hpi) with or without C3aRA treatment. Intracellular viral RNA levels (A) and the mRNA expression levels of the proinflammatory cytokines IL-1β (B) and IL-6 (C) were quantified by RT‒qPCR. Data are presented as mean ± SD. Statistical significance was determined by two-sided Dunnett’s multiple comparisons test following one-way ANOVA. *p < 0.05, **p < 0.01, ***p < 0.001.
(TIF)

S6 Fig Complement inhibition using a C3aR antagonist decreased the inflammatory response in IFNAR−/− mice after SFTSV challenge. I) *IFNAR*^*−/−*^ mice were challenged with SFTSV and then administered the C3aR antagonist (C3aRA) or the vehicle control. The mRNA expression levels of the cytokines IL-6, TNF-α, and IFN-γ in the lung, kidney, and liver tissues were quantified 3 days post SFTSV infection (n = 5 per group). Data are presented as the mean ± SD. Two-sided *p*-values, examined by Student’s t-test, are shown. *p < 0.05, **p < 0.01, ***p < 0.001.
(TIF)

S1 Table Detailed sequence information of siRNA and primers. (XLSX)

S2 Table Raw data of figures. (XLSX)
