# Supplementary figures and images for: C/EBP-β-regulated complement hyperactivation in spleen of SFTSV-infected mice: A clue to targeted complement therapy
Source: PLoS Pathog. 2026 Apr 15;22(4):e1014144. doi: 10.1371/journal.ppat.1014144 (PMC13128106; doi:10.1371/journal.ppat.1014144)

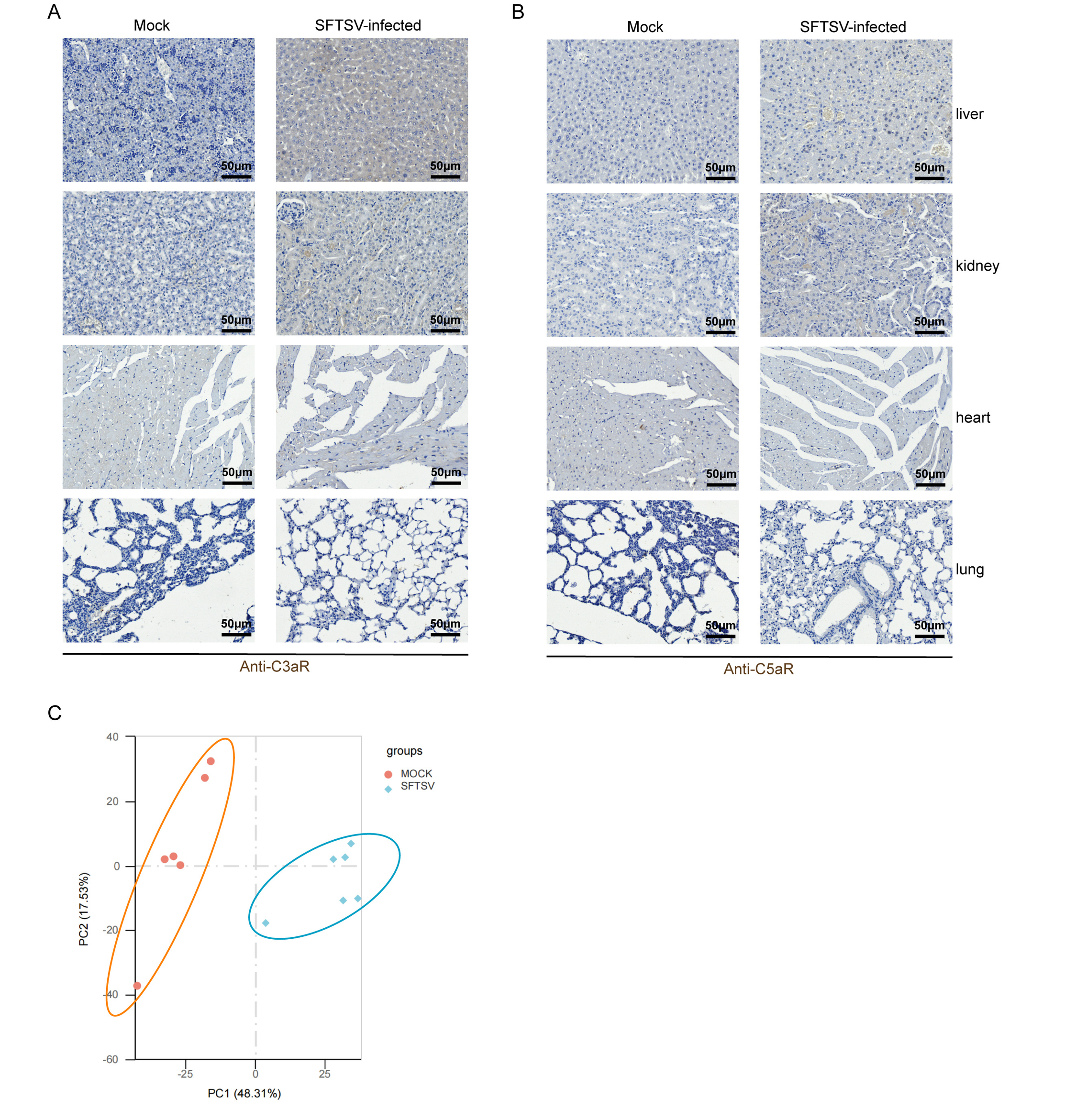

Supplement: S1 Fig — (A and B) Immunohistochemical staining for C3aR (A) and C5aR (B) in the liver, kidney, heart, and lung tissues. Mice were sacrificed and tissues were collected 3 days after SFTSV infection. (C) PCA of transcriptomics data in mock-infected (n = 6) and SFTSV-infected (n = 6) spleens. (TIF) [file ppat.1014144.s001.tif]

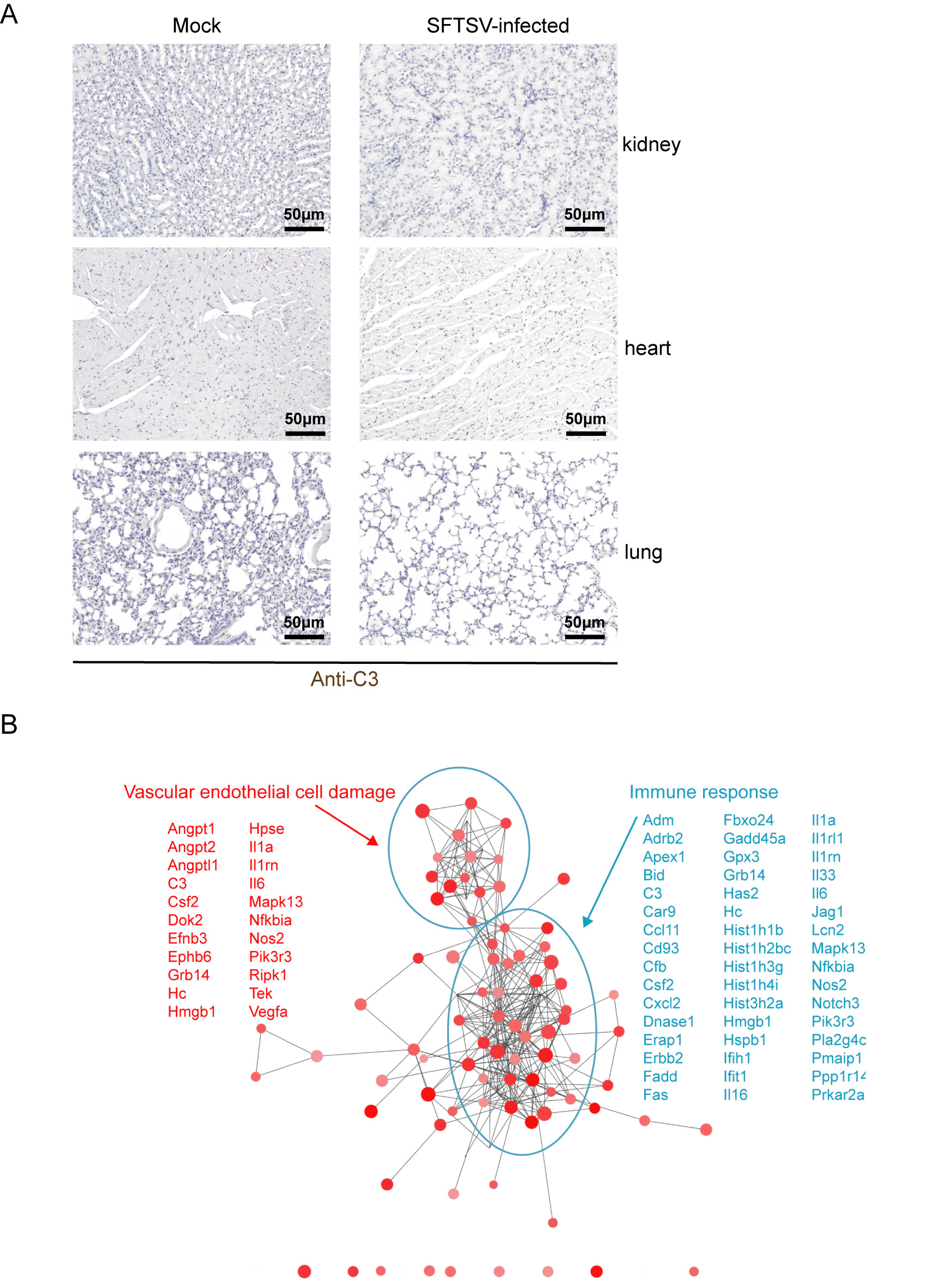

Supplement: S2 Fig — Immunohistochemical staining for C3 in the kidney, heart, and lung tissues. Representative images of immunohistochemical staining from ≥3 independent experiments are shown. (B) Significantly enriched biological processes analyzed by Metacore using DEGs between SFTSV-infected and mock-infected FRCs. The Enrichment Map application in Cytoscape was used for visualization. Nodes represent biological process terms. The color and size of the nodes reflect the significance of the terms and the number of objects enriched in the terms, respectively. The connection between terms is based on shared objects. (TIF) [file ppat.1014144.s002.tif]

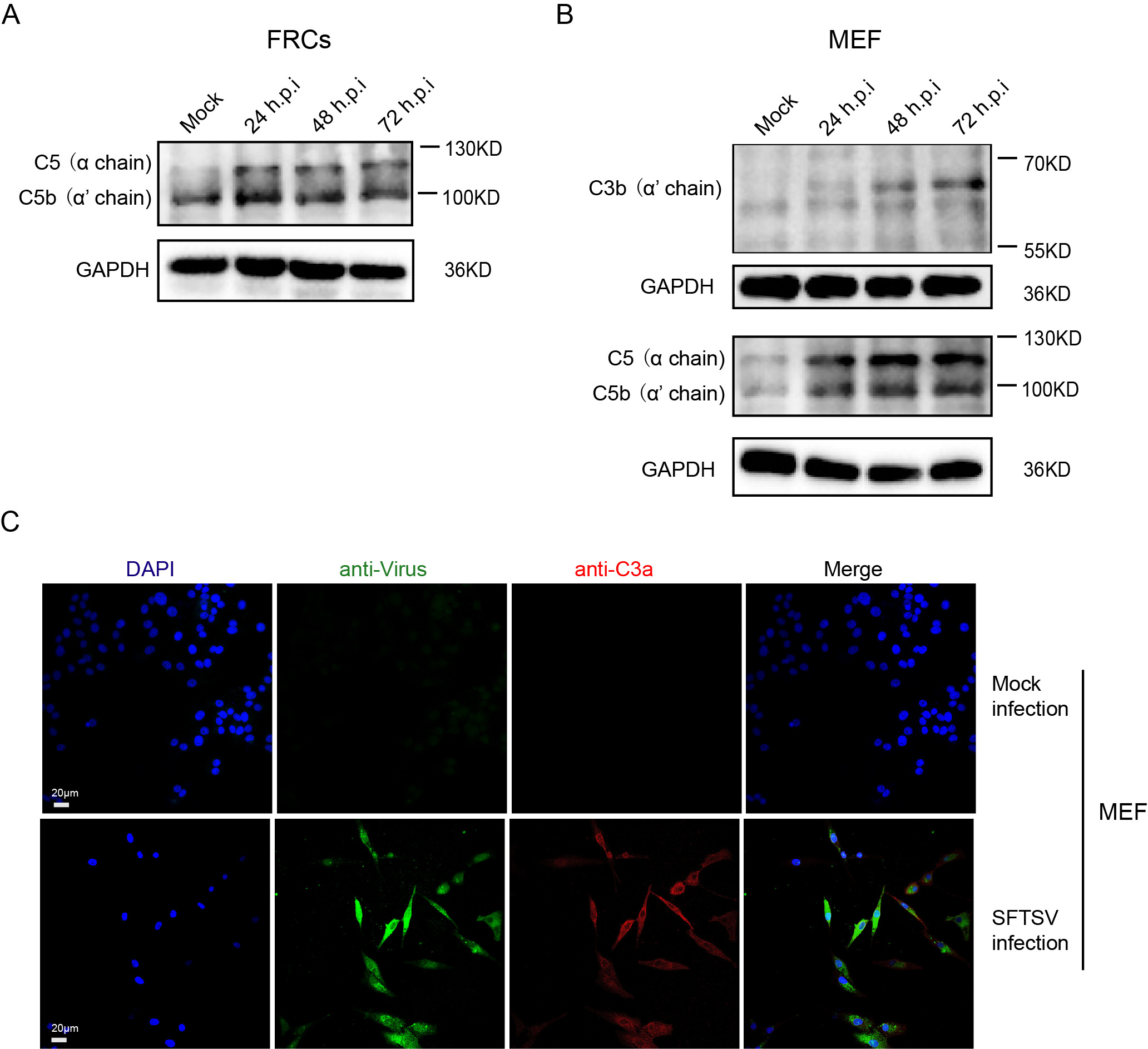

Supplement: S3 Fig — Time-course expression of C5b in FRCs at the indicated time points following SFTSV infection. (B) Time-course expression of C3b and C5b in MEFs at the indicated time points following SFTSV infection. (C) Representative confocal microscopy images showing the expression of C3a and SFTSV antigen in SFTSV-infected or mock-infected MEFs. n = 3 independent experiments. Scale bars are indicated in the images. (TIF) [file ppat.1014144.s003.tif]

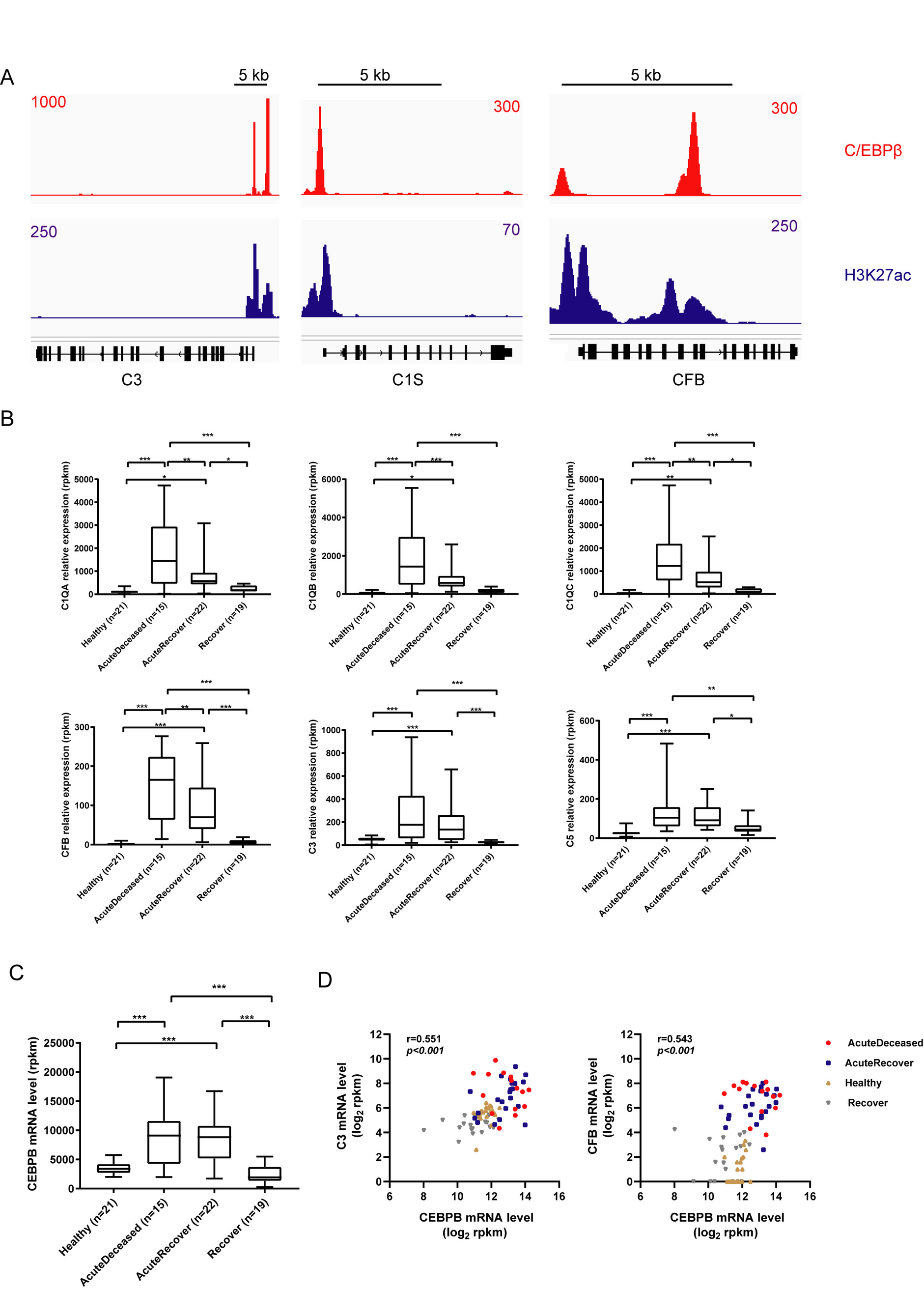

Supplement: S4 Fig — C/EBP-β and H3K27Ac ChIP-seq tracks showing the C3, C1S, and CFB gene loci. ChIP-seq data were sourced from ENCODE (H3K27Ac and C/EBP-β). (B-D) Transcriptome data of peripheral blood mononuclear cells (PBMCs) from healthy controls and SFTS patients were sourced from GSE144358. SFTS patients were stratified into three groups according to their clinical status. “Recover”: SFTS patients in the convalescent phase, who had recovered from acute viral infection; “AcuteRecover”: SFTS patients in the acute phase of viral infection who ultimately achieved recovery; and “AcuteDeceased”: SFTS patients in the acute phase of viral infection who eventually succumbed to SFTSV infection. (B) The mRNA expression levels of complement-related genes, including C1QA, C1QB, C1QC, C3, CFB, and C5, in PBMCs from healthy controls and SFTS patients. (C) C/EBP-β mRNA expression in PBMCs from healthy controls and SFTS patients. Box-and-whiskers plot features: whiskers represent the lowest and greatest values; boxes represent the median with the 25th percentile and the 75th percentile. (D) Correlation between the mRNA expression of C3 and CFB and the mRNA expression of C/EBP-β in PBMCs from healthy controls and SFTS patients. Indicated are the Pearson correlation coefficients and the associated p-values. Two-sided p-values, examined by Tukey’s multiple comparisons test after one-way ANOVA (B and C), are shown. *p < 0.05, ***p < 0.001. (TIF) [file ppat.1014144.s004.tif]

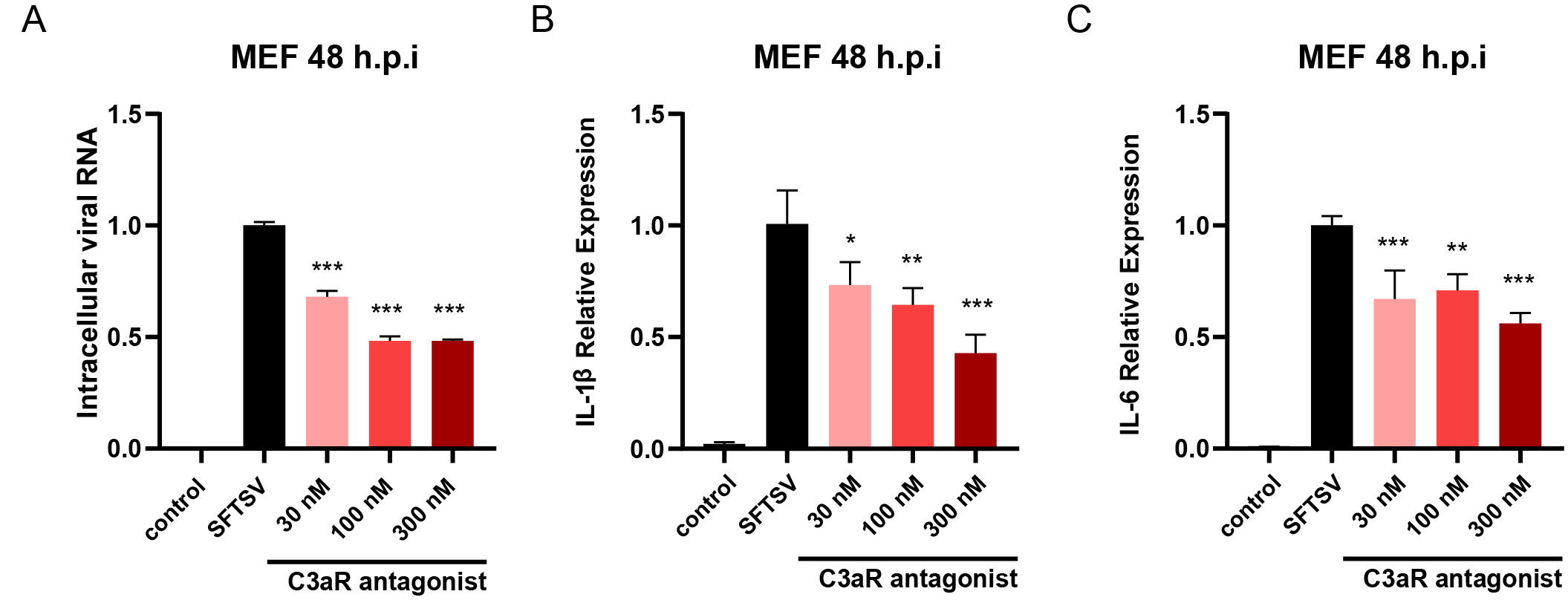

Supplement: S5 Fig — (A-C) MEFs were mock-infected or SFTSV-infected (1.0 MOI, 48 hpi) with or without C3aRA treatment. Intracellular viral RNA levels (A) and the mRNA expression levels of the proinflammatory cytokines IL-1β (B) and IL-6 (C) were quantified by RT‒qPCR. Data are presented as mean ± SD. Statistical significance was determined by two-sided Dunnett’s multiple comparisons test following one-way ANOVA. *p < 0.05, **p < 0.01, ***p < 0.001. (TIF) [file ppat.1014144.s005.tif]

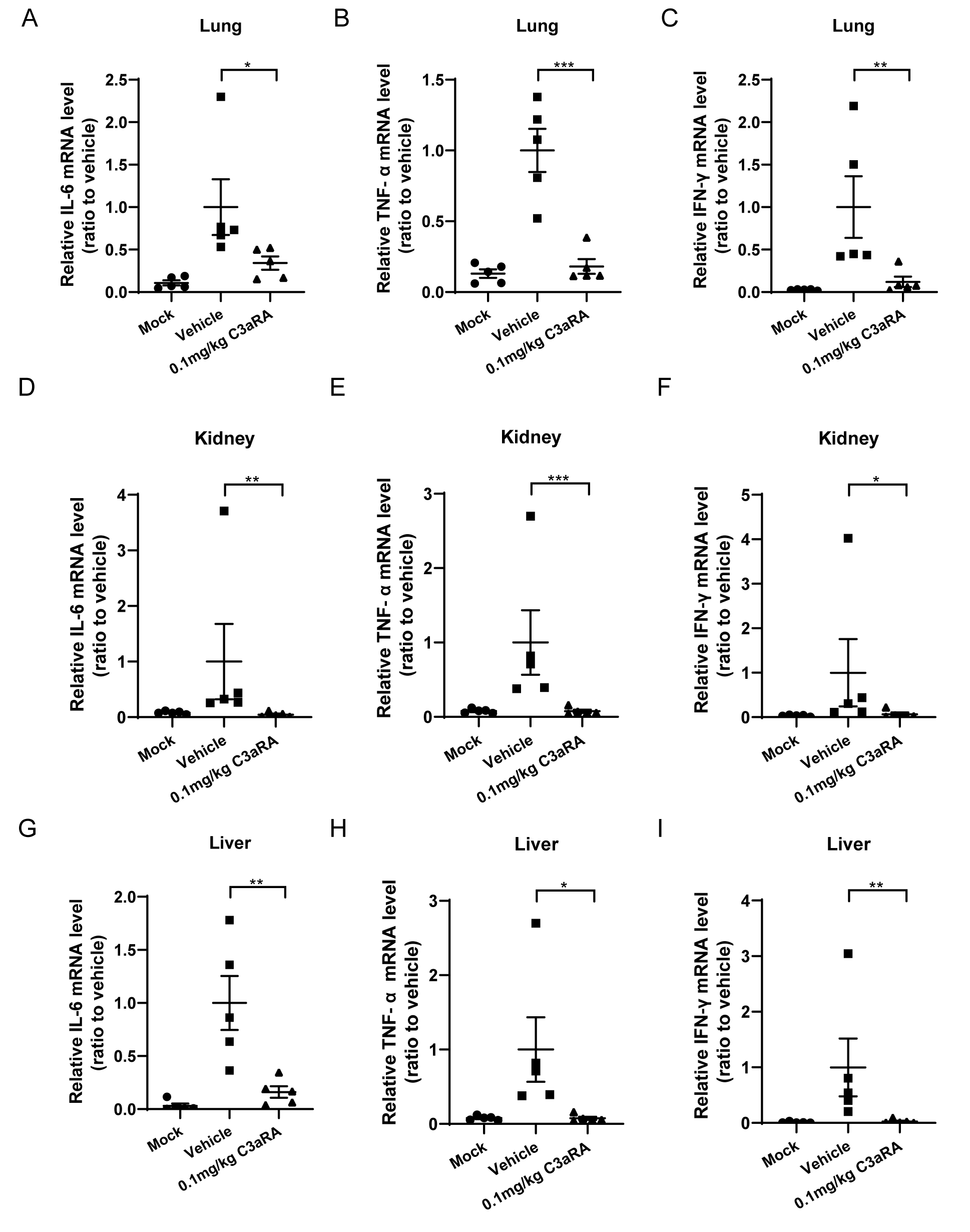

Supplement: S6 Fig — I) IFNAR−/− mice were challenged with SFTSV and then administered the C3aR antagonist (C3aRA) or the vehicle control. The mRNA expression levels of the cytokines IL-6, TNF-α, and IFN-γ in the lung, kidney, and liver tissues were quantified 3 days post SFTSV infection (n = 5 per group). Data are presented as the mean ± SD. Two-sided p-values, examined by Student’s t-test, are shown. *p < 0.05, **p < 0.01, ***p < 0.001. (TIF) [file ppat.1014144.s006.tif]
